# Multiglandular Hormone Deficiency in a Patient with Systemic Capillary Leak Syndrome

**DOI:** 10.1155/2015/958283

**Published:** 2015-01-05

**Authors:** Cornelia Then, Katrin Ritzel, Christa Seibold, Johannes F. E. Mann, Martin Reincke

**Affiliations:** ^1^Medizinische Klinik und Poliklinik IV, Klinikum der Universität München, Ziemssenstrasse 1, 80336 Munich, Germany; ^2^Klinik für Nephrologie, Klinikum Schwabing, Kölner Platz 1, 80804 Munich, Germany

## Abstract

Systemic capillary leak syndrome (SCLS) is a rare but potentially fatal disorder characterized by a loss of fluid and proteins into the interstitial space leading to intravascular hypovolemia up to the point of hypovolemic shock. We report the case of a 64-year-old man with SCLS and multiple hormone abnormalities (primary hypothyroidism, hypoadrenalism, and hypogonadism), deficiency of hormone binding globulins, and hypogammaglobulinemia. The patient was successfully treated with intravenous immunoglobulins, theophylline, and terbutaline. Strikingly, with the dissolution of peripheral edema, hormone levels improved. To our knowledge, this is the first reported case of SCLS associated with polyglandular abnormalities.

## 1. Introduction

Idiopathic systemic capillary leak syndrome (SCLS) is a rare but potentially life-threatening disease. Caused by a so far unexplained leakage of proteins and fluid from the intravascular into the interstitial space with subsequent formation of edema and intravascular hypovolemia, it is characterized by attacks of hypovolemic shock with variable intensity. Complications include venous or arterial thrombosis due to haemoconcentration, elevation of intracompartmental pressure followed by rhabdomyolysis, and hypoperfusion leading to acute organ failure primarily affecting the kidney [1, 2]. The 5-year survival rate is estimated to be 70% [3]. Due to the rareness of the disease, pathogenesis and optimal treatment of SCLS are ill defined and misdiagnosis or delayed diagnosis is frequent and may severely affect the patient's outcome. For instance, a protracted lag time of a median of 13.5 months was reported in a study including 25 SCLS patients [4].

Here, we present the case of a patient with SCLS associated with multiple hormone abnormalities and hypogammaglobulinemia. Not only SCLS but also hormone deficiency and hypogammaglobulinemia resolved after initiation of treatment with intravenous immunoglobulins, theophylline, and terbutaline.

## 2. Case Report

A 64-year-old man was admitted to the emergency room of our hospital with sudden onset of massive edema of the legs and arms, weight gain of 12 kg, anasarca, pleural and pericardial effusion, and anuria. At presentation, the patient had a heart rate of 110/minute, a systolic and diastolic blood pressure of 105 mmHg and 50 mmHg, a body weight of 87 kg, and flat external jugular veins at 10 degrees. The haemoglobin value was 19.5 g/dL, haematocrit 58%, serum albumin 3.3 g/dL (range 3.5–5.0 g/dL), and total protein level 5.2 g/dL (6.0–8.5 g/dL). The patient had three similar episodes of sudden onset edema during the preceding two months leading to hospital stays. The maximum haemoglobin value during these previous hospital stays had been 22 g/dL with a haematocrit of 65%. The patient had no further medical history or regular medication. During the previous attacks, he was treated with corticosteroids once for a short time.

Plasma creatinine was increased to 115 *μ*mol/L (44.2–97.2 *μ*mol/L). Urine protein and albumin ranged below 200 mg/g creatinine and urine sodium between 90 and 160 mmol/g creatinine. The leukocyte count was elevated to 28,400/*μ*L with 91% neutrophils and 6% lymphocytes, whereas C-reactive protein was only slightly elevated to 51.4 nmol/L (<47.6 nmol/L). Complement factor C3 was decreased to 4.5 *μ*mol/L (4.73–9.47 *μ*mol/L). Hepatitis B or C infection, tuberculosis, alpha-1-antitrypsin deficiency, Budd-Chiari syndrome, hepatic, renal, or cardiac failure, protein-losing enteropathy, lymphatic and venous disorders, postdiuretic abuse, and excessive sodium chloride intake were largely excluded. An immunoglobulin deficiency with decreased levels of IgG, IgA, and IgM (Table 1) was apparent. Serum immune electrophoresis displayed a minimal monoclonal IgG kappa gammopathy, though bone marrow biopsy revealed no monoclonal B cells or plasma cells.

Because of the obscure clinical presentation and in order to rule out endocrine causes of unexplained shock, such as adrenal failure, the patient underwent detailed endocrine testing. Surprisingly, several hormone levels were altered: TSH, LH, and FSH were increased, whereas fT3 and fT4 were normal, and testosterone and free androgen index were decreased, indicating subclinical primary hypothyroidism and manifest primary hypogonadism. Anti-thyroperoxidase antibodies and anti-thyroglobulin antibody titers were normal. Furthermore, the patient suffered from adrenal insufficiency with an insufficient cortisol rise after ACTH stimulation and a reduced dehydroepiandrosterone sulphate (DHEAS) level, whereas ACTH was normal or elevated. Prolactin, hGH, and IGF-1 were in the standard range. Thyroxine-binding globulin (TBG) and transcortin were reduced, whereas sex hormone-binding globulin (SHBG) was elevated. Renin and aldosterone were in a high range. This unique pattern of hormone abnormalities did not resemble the changes seen in critical illness [5] (Table 1).

**Figure 1:**
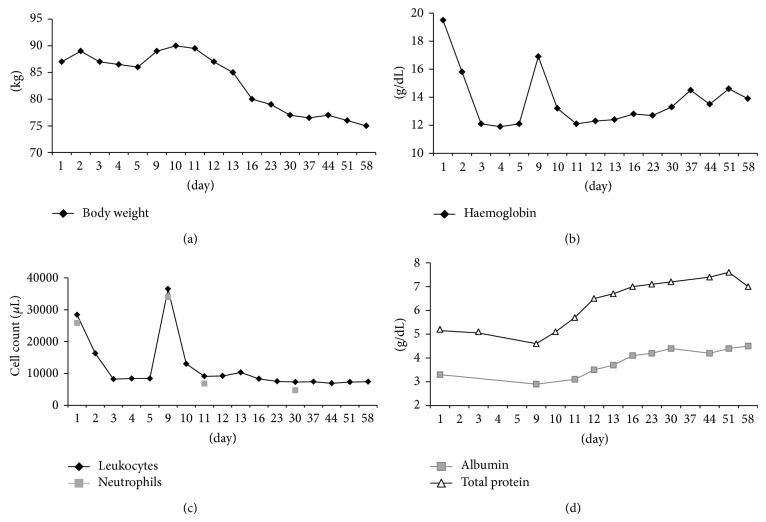
Time course of body weight, haemoglobin, leukocyte and neutrophil counts, and albumin and total protein levels of the described patient. SCLS attacks occurred on days 1 and 9.

Due to the presence of the characteristic triad of hypotension, haemoconcentration, and hypoalbuminemia, the patient was diagnosed with idiopathic SCLS and treated with intravenous 0.9% sodium chloride solution to restore the intravascular fluid balance. Prednisolone 100 mg daily orally was started and thyroxine was substituted. The change of body weight, haemoglobin, albumin and total protein levels, and leukocyte and neutrophil counts in response to treatment is shown in Figure 1. After intravenous fluid substitution, body weight initially increased but started to decrease on the third day. Haemoconcentration and leukocytosis resolved during the next four days. The patient was discharged at day 5 on prednisolone therapy of 60 mg daily and thyroxin 100 *μ*g daily. Four days later, the patient represented due to rapidly increasing peripheral edema, hypotension, and anuria. As shown in Figure 1, body weight, haemoglobin level, and leukocyte count had increased again. Prednisolone was stopped and the patient was treated with 30 g intravenous immunoglobulins on days 9 and 10. Theophylline with an intended plasma level of 10–20 *μ*g/mL and terbutaline at a dose of 7.5 mg 3 times daily were started. Intravenous immunoglobulin therapy was repeated at a dose of 60 g at day 33.

During the following month, body weight, haemoglobin level, leukocyte counts, albumin and total protein values (Figure 1), serum complement factor C3, and creatinine normalized. Moderate edema was still present for several weeks but eventually resolved. Interestingly, also IgG and IgM reached a normal level at week 8. The hormone deficiencies resolved nearly completely during the further disease course (Table 1) and thyroxin substitution was terminated at week 11. Serum cortisol levels after ACTH 1-24 stimulation remained subnormal, and plasma ACTH levels were elevated indicating persistent subclinical primary adrenal insufficiency. Since the patient was free of symptoms of Addison's disease, glucocorticoid replacement therapy was withheld. The patient did not experience another SCLS attack during the follow-up time of more than 24 months.

## 3. Discussion

We describe a patient with idiopathic SCLS who in parallel with SCLS episodes displayed multiple hormone deficiencies and strikingly elevated leukocyte and particularly neutrophil counts. Further features were hypogammaglobulinemia and monoclonal gammopathy of unclear significance (MGUS).

The pathogenic mechanisms of SCLS are still obscure. An association with single nucleotide polymorphisms implies a genetic background of SCLS [6]. The origin of the increased susceptibility to vascular hyperpermeability is thought to lie in serum factors, not in the vasculature itself, as endothelial cadherin internalization and disruption of interendothelial junctions with subsequent increased permeability were inducible in human microvascular endothelial cells by exposure to sera from SCLS patients [7]. Several factors mediating the increased vascular permeability have been proposed, primarily cytokines, such as interleukins 1*β*, 2, 6, 8, and 12, interferons gamma and alpha, tumor necrosis factor alpha, vascular endothelial growth factor, and C-X-C motif chemokine 10 and chemokine (C-C motif) ligand 2, but also various other factors, for example, epoprostenol, gemcitabine, hepatitis C-infections, and malignancies [8–13].

Granulocyte colony stimulating factor (G-CSF), irrespectively of whether the serum level rises idiopathically or after therapeutic administration, may promote endothelial damage by increasing the production of granulocytes and the release of cytokines and superoxide anion radicals [12–15]. In the present case, neutrophil counts were strongly elevated during the attacks, supporting this hypothesis. Monoclonal serum paraprotein, mainly relatively small amounts of IgG kappa, is observed in approximately 80% of SCLS patients and is the only persistent laboratory abnormality between attacks [1, 4]. However, attempts to directly link the paraprotein to endothelial damage have been unsuccessful, since no immunoglobulin deposits were detected [2] and purified monoclonal paraprotein from five SCLS patients failed to bind cultured endothelial cells [11, 16, 17]. Yet, unlike purified paraprotein, serum from SCLS patients can induce apoptosis in cultured endothelial cells [18]. Thus, it is speculated that a toxic monoclonal paraprotein may bind and inhibit a so far unidentified factor crucial for endothelial barrier function [2], which may provide the premise for SCLS. Considering elevated cytokine levels and increased numbers of circulating CD25^+^ cells reported in several cases [19–21], perivascular mononuclear infiltrates in skin biopsies taken during attacks [14, 22], and the described elevated G-CSF levels and neutrophil counts, a general factor initiating SCLS attacks may be a so far unspecified immune dysregulation.

Although proteins with weights up to 900 kDa may extravasate during an SCLS attack [4], deficiency of hormones or hormone-binding proteins exhibiting molecular weights far below 900 kDa has to our knowledge not yet been reported. We found reduced levels of testosterone, cortisol after ACTH stimulation, DHEAS, TBG, and transcortin, whereas TSH, LH, and FSH were upregulated, indicating primary hypothyroidism, hypogonadism, and adrenal insufficiency. We speculate that hormone-binding globulins and hormones were lost into the interstitial space along with other proteins due to an extensive vascular leak. This hypothesis is supported by the fact that the relevant protein concentrations returned to normal or near-normal after resolution of SCLS attacks and no long-term signs or symptoms of hormone deficiencies were present. Primary hypothyroidism and low TBG (54 kDa) were detected early on and resolved in response to treatment of SCLS.

Dysfunction of the adrenal and gonadal axis appears to be more complex. SHBG with a molecular weight of 86 kDa was elevated, but total and free testosterone were markedly reduced and recovered only slowly despite elevated LH concentrations. Pituitary-adrenal function was impaired initially and did not completely recover during follow-up. Transcortin was initially decreased and remained close to the lower value of the normal range during follow-up. Renin and aldosterone were in a high range, probably provoked by intravascular hypovolemia. Due to a lower degree of binding to plasma proteins, aldosterone may not have been affected by the loss of plasma proteins the same way as cortisol. High aldosterone levels furthermore affirm that, in this case, adrenal deficiency was not caused by a primary synthesis defect of the adrenal cortex.

By chance, the pattern of hormone-binding protein deficiency hints at the size of proteins leaking into the interstitial space: contrary to TBG (54 kDa) and transcortin (52 kDa), SHBG (86 kDa) levels were elevated, which might give insight into respective permeability of the endothelial barrier. On the other hand, immunoglobulins with much higher molecular weights (144 to 971 kDa) were also decreased. Although described previously [23], immunoglobulin deficiency is not a common finding in SCLS. If it is hypothesized that the antibody deficiency in the present patient was caused by immunoglobulin loss due to the vascular leakage, a severe vascular damage may be assumed, since especially IgM is a large molecule with a molecular weight of 971 kDa. However, the fact that during the further course immunoglobulin levels dropped without the presence of signs of SCLS suggests that the decreased antibody levels were rather an expression of an immune dysfunction involved in the pathogenesis of SCLS. The observation that the patient developed no further SCLS attack despite falling antibody values may be attributable to the continued treatment.

Standard treatment of SCLS is not yet established. Corticosteroids interfere with granulocyte function and cytokine release and are occasionally successful in reducing the severity but do not always prevent attacks from progressing [1, 2, 4, 7] and were of no benefit in our patient, who suffered early relapse during on-going prednisolone therapy. Immunosuppressive or immunomodulating drugs, such as infliximab, bevacizumab, and thalidomide, proved helpful in some SCLS cases [20, 24, 25]. However, the most successful therapeutic measure during an attack to date is the application of intravenous immunoglobulin [2, 4, 26–29], which the described patient received at a total dose of 120 g. For prevention of new SCLS attacks, administration of theophylline, which inhibits cyclic adenosine monophosphate degradation, and terbutaline, a beta-adrenergic agonist increasing cyclic adenosine monophosphate, is the most established therapy to reduce the frequency and severity of SCLS attacks [4] and proved to be successful in the described patient until today. Regarding the various hormone deficiencies, we temporarily substituted thyroxine. We refrained from glucocorticoid replacement, since the patient was lacking signs and symptoms of adrenal insufficiency. In conclusion, although we cannot prove a direct pathophysiological connection of the hormonal changes and SCLS, clinicians have to be aware that SCLS may be accompanied by endocrine dysfunction in a degree that might be sufficient to necessitate hormone replacement therapy.

## Figures and Tables

**Table 1:** Hormone, hormone-binding globulin, and immunoglobulin levels of the described patient at the indicated time points after first admission to our hospital due to a systemic capillary leak attack. For comparison, commonly observed hormonal changes in critically ill patients are shown.

Parameter	kDa	Range	Day 2	Day 12	Day 23	Week 8	Week 11	Week 18	Month 10	Critical illness (acute)	Critical illness (prolonged)
IgG (µmol/L)	144	55.5–125	**46.9**	—	83.3	109.9	—	**52.4**	**49**		
IgA (µmol/L)	160	5.6–28	**2.4**	—	**4.1**	**4.8**	**—**	**3.9**	**4.3**		
IgM (µmol/L)	971	0.6–2.6	**0.4**	—	0.7	0.8	—	**0.5**	0.6		
TSH (mU/L)	28	0.3–4	**6.1**	—	—	—	2.01	2.03	2.41	→ (↓)	↓
fT3 (pmol/L)	0.65	3.5–6.6	5.8	—	—	—	—	—	6.1	↓	↓
fT4 (pmol/L)	0.8	10.3–24.5	13.7	—	—	—	—	—	17.4	→ (↓)	→ (↓)
LH (U/L)	24	1.2–8	**13.5**	**—**	**13.8**	—	—	—	7.4	↑	↓
FSH (U/L)	32	1–9	**14.4**	**—**	**15.0**	**—**	**—**	**—**	**15.3**	→ (↑)	↓
ACTH (pmol/L)	4.5	2.2–15.4	5.5	—	—	—	**17.8**	16.1	**21.6**	↑	↓
hGH (nmol/L)	22	0–360	16.3	—	—	—	—	—	47	↑	(↑)
IGF-1 (nmol/L)	7.6	9.2–38	12.9	—	—	—	—	—	25	↓	↓
Prolactin (mU/L)	22.9	30–350	261	—	—	—	—	—	—	↑	(↓)
Testosterone (nmol/L)	0.29	12–35	**3.8**	—	14.3	—	**10.8**	**10.0**	14	↓	↓
Free androgen index (%)	—	>22	**5.2**	**—**	**17.7**	**—**	**17.2**	**12.4**	30	↓	↓
Cortisol after ACTH stimulation (nmol/L)	0.36	635–1560	**333.8**	**—**	**460.7**	**474.5**	**430.4**	**—**	**422**	↑↑	↑↑
DHEAS (µmol/L)	0.37	0.95–11.7	**0.54**	—	—	1.63	—	1.64	—	↓	↓↓
Transcortin (µmol/L)	52	0.8–1.1	—	**0.6**	0.8	**0.7**	—	—	—	↓	↓
TBG (nmol/L)	54	260–500	—	—	**250**	330	—	—	—	↓	↓
SHBG (nmol/L)	86	10–50	70.5	—	81.3	—	58.2	58.3	**47**	↓	↓
Aldosterone (pmol/L)	0.36	55–416	—	368	**512**	**867**	—	304	**450**	→↑	
Plasma renin (mU/L)	42		—	309	245	1250	—	137	202	↑↑	
